# Decreased pericellular matrix production and selection for enhanced cell membrane repair may impair osteocyte responses to mechanical loading in the aging skeleton

**DOI:** 10.1111/acel.13056

**Published:** 2019-11-19

**Authors:** Mackenzie L. Hagan, Kanglun Yu, Jiali Zhu, Brooke N. Vinson, Rachel L. Roberts, Marlian Montesinos Cartagena, Maribeth H. Johnson, Liyun Wang, Carlos M. Isales, Mark W. Hamrick, Paul L. McNeil, Meghan E. McGee‐Lawrence

**Affiliations:** ^1^ Department of Cellular Biology and Anatomy Medical College of Georgia Augusta University Augusta GA; ^2^ Department of Neuroscience and Regenerative Medicine Augusta University Augusta GA; ^3^ Department of Mechanical Engineering University of Delaware Newark DE; ^4^ Department of Orthopaedic Surgery Augusta University Augusta GA

**Keywords:** aging, bone, mechanical loading, mechanotransduction, osteocyte, skeleton

## Abstract

Transient plasma membrane disruptions (PMD) occur in osteocytes with in vitro and in vivo loading, initiating mechanotransduction. The goal here was to determine whether osteocyte PMD formation or repair is affected by aging. Osteocytes from old (24 months) mice developed fewer PMD (−76% females, −54% males) from fluid shear than young (3 months) mice, and old mice developed fewer osteocyte PMD (−51%) during treadmill running. This was due at least in part to decreased pericellular matrix production, as studies revealed that pericellular matrix is integral to formation of osteocyte PMD, and aged osteocytes produced less pericellular matrix (−55%). Surprisingly, osteocyte PMD repair rate was faster (+25% females, +26% males) in osteocytes from old mice, and calcium wave propagation to adjacent nonwounded osteocytes was blunted, consistent with impaired mechanotransduction downstream of PMD in osteocytes with fast PMD repair in previous studies. Inducing PMD via fluid flow in young osteocytes in the presence of oxidative stress decreased postwounding cell survival and promoted accelerated PMD repair in surviving cells, suggesting selective loss of slower‐repairing osteocytes. Therefore, as oxidative stress increases during aging, slower‐repairing osteocytes may be unable to successfully repair PMD, leading to slower‐repairing osteocyte death in favor of faster‐repairing osteocyte survival. Since PMD are an important initiator of mechanotransduction, age‐related decreases in pericellular matrix and loss of slower‐repairing osteocytes may impair the ability of bone to properly respond to mechanical loading with bone formation. These data suggest that PMD formation and repair mechanisms represent new targets for improving bone mechanosensitivity with aging.

## Graphical abstract

Pericellular matrix (blue) decreases during aging, which limits the number of membrane disruptions that form from a given load. Slower‐repairing osteocytes progressively succumb to repair failure from increasing oxidative stress during aging whereas rapidly repairing cells survive. Fast repair of membrane disruptions in surviving cells blunts their ability to propagate downstream mechanotransduction. Impaired formation of membrane disruptions during loading and rapid repair of membrane disruptions that do form may contribute to impaired bone adaptation in aged subjects.

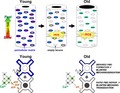

## 1 INTRODUCTION

Adaptation to mechanical loading is important for maintaining bone health throughout life, and impairments to bone adaptation responses, such as with aging, can contribute to diseases like osteoporosis. Previous studies indicate that bone's mechanosensing osteocytes are lost with aging (Galea et al., 2017; Mullender, van der Meer, Huiskes, & Lips, 1996; Tiede‐Lewis et al., 2017), and that surviving osteocytes may have impaired mechanotransduction responses (Chalil et al., 2015; Donahue, Jacobs, & Donahue, 2001; Holguin, Brodt, & Silva, 2016). Accordingly, while mechanical loading of bone via exercise is an effective method to increase peak bone mass in younger individuals (Greene et al., 2005; Kontulainen, Sievanen, Kannus, Pasanen, & Vuori, 2003; Warden et al., 2017; Weatherholt & Warden, 2018), exercise‐based approaches produce modest effects (at best) on bone properties in elderly subjects (Gomez‐Cabello, Ara, Gonzalez‐Aguero, Casajus, & Vicente‐Rodriguez, 2012; Karlsson, 2002). Young mice subjected to treadmill exercise showed significant suppression of sclerostin and RANKL/OPG ratio and increases in bone mechanical properties, whereas older mice subjected to an identical exercise regimen did not demonstrate substantial bone adaptation (Gardinier, Rostami, Juliano, & Zhang, 2018). Several causative factors for this blunted mechanical adaption phenomenon with aging have been suggested. For example, one study showed that aged cortical bone demonstrated an altered transcriptional response following mechanical loading, with a suppression of functional signaling cascades and a blunted activation timeframe downstream of loading as compared to loaded bones from younger subjects (Galea et al., 2017). Even in a sedentary state, aged cortical bone demonstrated an upregulation of TGF‐beta signaling and inflammatory TNF‐alpha signaling pathways, and a downregulation of Wnt pathway components and genes related to cell cycle progression (Galea et al., 2017). Aged bone required higher strains to trigger comparable adaptation to young bone, and transient application of a short burst of high magnitude loading was sufficient to prime‐aged cortical bone for proper adaptation (Javaheri et al., 2018). However, a mechanistic explanation for these observations has not yet been reported.

The precise mechanisms through which osteocytes sense mechanical stimuli are still under investigation, and as the mechanisms used by osteocytes for mechanosensation are not fully understood, the effects of aging on these processes are even less clear. It has been suggested that fluid drag forces on the osteocyte pericellular matrix (i.e., glycocalyx) are important for osteocyte mechanotransduction, and that fiber volume fraction in the osteocyte pericellular matrix is decreased in aged as compared to younger bone (Wang et al., 2014), but a direct relationship between osteocyte pericellular matrix and impaired mechanosensation with aging has not yet been demonstrated. We and others have shown that transient plasma membrane disruptions (PMD) develop in osteocytes with mechanical loading both in vitro (fluid flow) and in vivo (exercise), and that these PMD initiate mechanotransduction (Hagan et al., 2019; Mikolajewicz, Sehayek, Wiseman, & Komarova, 2019; Mikolajewicz, Zimmermann, Willie, & Komarova, 2018; Yu et al., 2018), suggesting that osteocytes may use PMD to detect mechanical loads and direct the activity of remodeling cells to properly adapt the matrix as needed. The effects of aging on osteocyte PMD formation, however, have not been reported. The goal of the current study was to assess whether osteocyte PMD formation, repair, and/or downstream PMD‐initiated mechanotransduction are affected by aging.

## 2 RESULTS

### 2.1 *Aged osteocytes develop fewer PMDs with mechanical loading both *in vitro* and *in vivo

**Figure 1:**
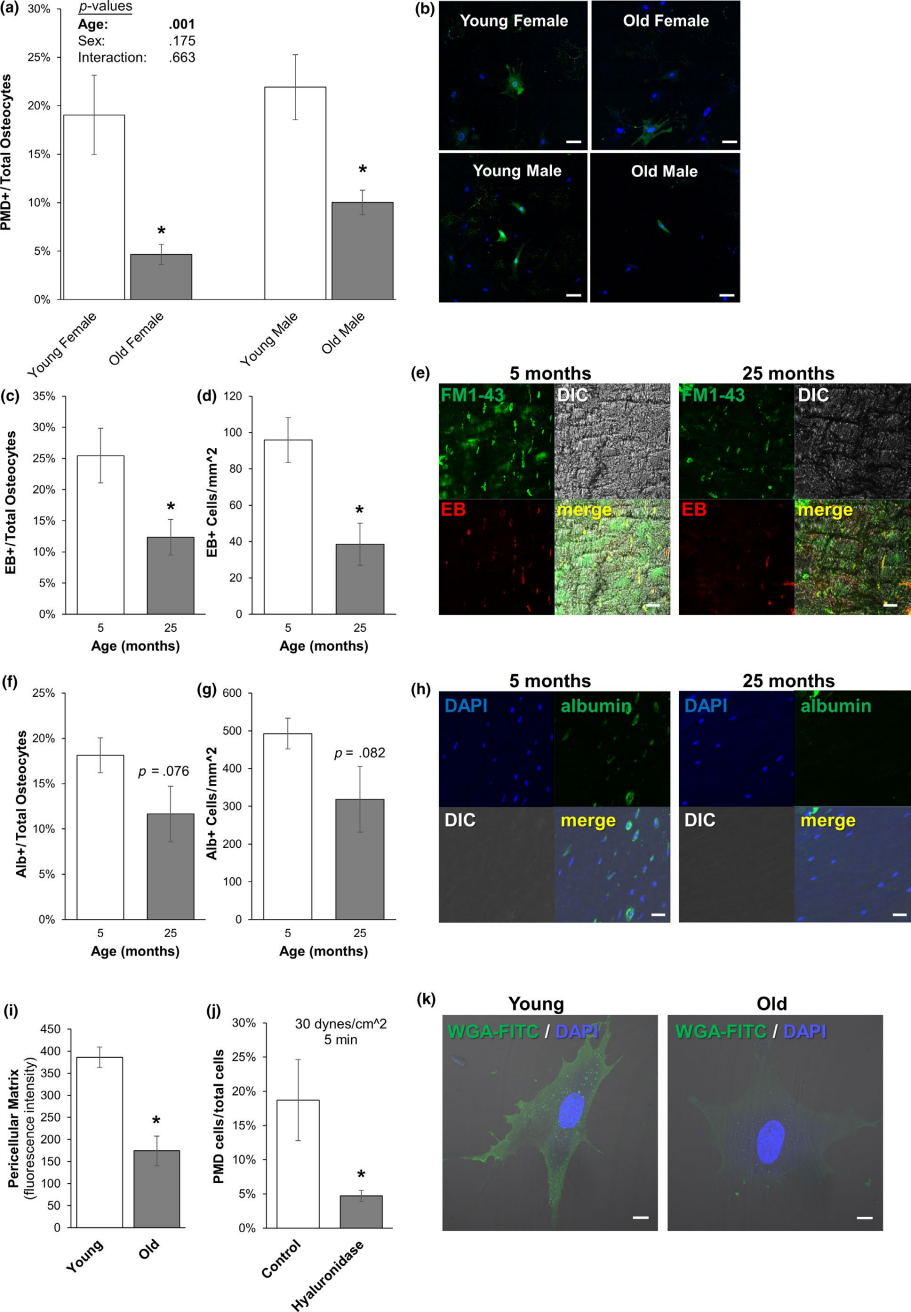
Effects of aging on PMD development in vitro and in vivo. (a–b) Primary osteocytes from old (24 months) male and female mice showed a significant reduction (−76% female, −54% male) in the development of PMD as compared to cells from young (3 months) mice when exposed to 30 dynes/cm^2^ fluid flow shear stress. *N* = 3 independent cell lines per age and sex. Representative images from each group are shown in panel b. *p < .05 versus sex‐matched young control. Scale bar: 100 µm. (c–e) Aged female mice showed a significantly lower percentage (−51%) and spatial density (−60%) of Evans Blue labeled osteocytes as compared to young controls. Representative images of Evans Blue labeling are shown in panel e. *p < .05 versus young control. Scale bar: 20 µm. (f–h) Aged female osteocytes showed a trend of reduced percentage (*p* = .076) and spatial density (*p* = .082) of albumin‐labeled osteocytes as compared to young controls with endogenous albumin staining. Representative images of albumin labeling are shown in Panel h. Scale bar: 20 µm. *N* = 4–5 mice per group. (i and k) Aged male primary osteocytes demonstrated significantly reduced pericellular matrix production in vitro as compared to young controls. *p < .05 versus Young. Representative images are shown in panel k. Scale bar: 10 µm. (j) MLO‐Y4 cells treated with hyaluronidase developed significantly fewer PMD following exposure to 30 dynes/cm^2^ as compared to vehicle‐treated control cells. *p < .05 versus vehicle‐treated control; average of *n* = 3 independent experiments

To determine the effects of aging on the formation of fluid shear‐induced PMD formation, old and young primary osteocytes were subjected to a fluid flow shear stress known to produce osteocyte PMD (30 dynes/cm^2^, 5 min) (Yu et al., 2018). Interestingly, aged osteocytes developed significantly fewer (−76% female, −54% male) PMD from fluid flow shear stress than young osteocytes for both males and female groups (Figure 1a,b). No differences were observed between the responses of male and female cells, and there was not a significant interaction between age and sex (Figure 1a,b).

To test this observation in vivo, old and young female mice were subjected to downhill treadmill running, which also induces the formation of PMD in long bone osteocytes (Hagan et al., 2019; Yu et al., 2018). Female mice were specifically tested to avoid confounding influence from fighting in group‐housed male mice (Meakin et al., 2013), although osteocyte PMD occur with treadmill loading in both male and female mice (Hagan et al., 2019; Yu et al., 2018). Similar to in vitro findings, old female mice demonstrated a lower percentage (−35% to –50%) and spatial density (−35% to −60%) of osteocytes with PMD in the femur or tibia caused by treadmill exercise as compared to young females, as measured by either intracellular endogenous mouse albumin or intracellular exogenous Evans Blue (Figure 1c–h), although immunostaining‐based measurements of albumin did not quite reach statistical significance (*p* = .08).

### 2.2 Aged osteocytes produce less pericellular matrix and have altered morphology, which may contribute to lower PMD formation

The fiber volume fraction of osteocytic pericellular matrix is decreased in aged as compared to younger bone (Wang et al., 2014). To test whether this phenomenon occurred in a cell‐intrinsic fashion and would be replicated in primary osteocyte cultures, osteocytes were grown for 4 days to permit growth of the glycocalyx and were stained with a lectin‐based reagent to detect pericellular matrix as previously described (Barker et al., 2004; Reilly, Haut, Yellowley, Donahue, & Jacobs, 2003). Similar to published in situ observations, primary osteocytes from aged mice demonstrated reduced production of pericellular matrix in vitro, as seen by a reduced signal for binding of carbohydrate‐binding lectins to the cell periphery (Figure 1i and Figure 1k). To test the effects of lower pericellular matrix on PMD formation, MLO‐Y4 osteocytes were treated with hyaluronidase, which removes the osteocyte pericellular matrix (Reilly et al., 2003). Hyaluronidase‐treated MLO‐Y4 cells developed significantly fewer membrane disruptions than vehicle‐treated cells during fluid flow, suggesting that pericellular matrix is important to the formation of membrane disruptions with fluid flow (Figure 1j). Therefore, it is possible that reduced pericellular matrix generation by aged osteocytes contributes to their lower incidence of membrane disruption formation when subjected to fluid shear.

As an additional mechanism possibility, it was recently reported that aged bone contains osteocytes with reduced dendritic connectivity and number compared with young bone in both males and females (Tiede‐Lewis et al., 2017), and osteocyte PMD tend to form on the dendrites (Yu et al., 2018). From morphological measurements of primary osteocytes in the current studies, we found that altered dendritic morphology with aging holds true in vitro as well, as aged osteocytes had fewer dendritic processes than osteocytes from young mice (Supporting Information Figure S1a), although this trend was more pronounced in osteocytes from female mice as compared to male mice, and a significant interaction effect between age and sex was observed for dendrite number (Supporting Information Figure S1a). Dendrite length was also significantly affected with age in a sex‐dependent manner, with aged male osteocytes demonstrating shorter processes and aged female osteocytes demonstrating longer processes than sex‐matched counterparts (Supporting Information Figure S1b). Aged primary osteocytes also tended to have smaller cell bodies than young osteocytes (Supporting Information Figure S1c; *p* = .091), which is consistent with previous observations from histological sections (Tiede‐Lewis et al., 2017).

### 2.3 Aged primary osteocytes repair membrane disruptions faster than young osteocytes, which may impair mechanotransduction

**Figure 2:**
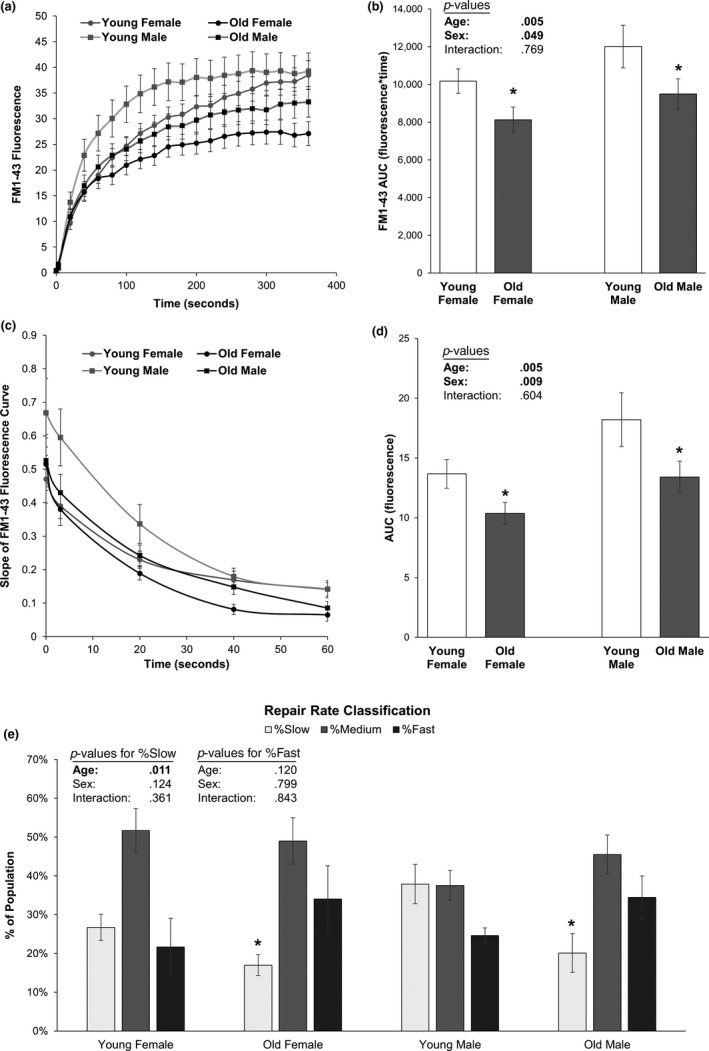
Effects of aging on osteocyte membrane repair rate. (a–b) Aged female and male osteocytes demonstrated significantly faster rates of membrane repair, as seen by a significant effect of age in 2‐factor ANOVA analyses (*p* = .005) for area under the curve (AUC) in old as compared to young cells. The AUC was slightly but significantly greater in male than in female osteocytes, suggesting a mildly faster repair rate in females than in males (*p* = .049). *p < .05 versus sex‐matched young control. (c–d) The slopes of the fluorescence versus time curves presented in panel a were calculated and plotted during the first 60 s of PMD repair to directly quantify repair rate; these data also suggested significantly faster rates of membrane repair in old as compared to young osteocytes, as seen by a significant effect of age in 2‐factor ANOVA analyses (*p* = .005) for AUC. *p < .05 versus sex‐matched young control. (e) Repair rate at 60 s after wounding was quantified and used to stratify individual osteocytes into “fast,” “medium,” and “slow” repairing categories, where fast‐repairing osteocytes had repair curve slopes below the lower quartile of the dataset and slow‐repairing osteocytes had repair curve slopes greater than the upper quartile of the dataset. Aged male and female osteocyte cultures had significantly fewer slow‐repairing cells (*p* = .011) as compared to young cultures. *N* = 3 independent cell lines per age and sex. *p < .05 versus sex‐matched young control for each category

Aged bone is less mechanosensitive than young bone, and temporal delay of wound repair is a common feature of aging (Gosain & DiPietro, 2004; Keylock et al., 2008; McBride, 2000; McBride, Gorin, & Carlsen, 1995; Naik et al., 2009). Increasing numbers of vacant osteocyte lacunae (implying osteocyte death) with age (Qiu, Rao, Palnitkar, & Parfitt, 2002), correlations between loss of osteocytes and development of an aged and less mechanosensitive bone phenotype (Tatsumi et al., 2007), and observations of greater damage/slower repair in PMD‐affected muscle from aged as compared to young mice could be consistent with an aging‐related PMD repair defect in musculoskeletal tissues (McBride et al., 1995). Accordingly, we hypothesized that aged osteocytes would show slower membrane repair rates than younger cells. Unexpectedly, old osteocytes consistently demonstrated faster PMD repair rates in both males and females, as measured by a lower FM 1–43 fluorescence at each time point after wounding (Figure 2a,b) and by slopes of the FM 1–43 fluorescence curve that were less steep than young cells (Figure 2c,d). Area under the curve (AUC) from repair rate experiments was significantly decreased (indicating faster repair) in old as compared to young female (−20 to −24%) and male (−21 to −26%) osteocytes (Figure 2a–d), with no interaction effects between sex and age (*p* = .769). The AUC for the fluorescence versus time curve and the slope versus time curve was lower in osteocytes from females than in males, suggesting faster repair rates in female osteocytes (Figure 2a–d; *p* < .049). Primary osteocyte cultures from old mice contained significantly fewer slow‐repairing osteocytes (−36% female, −47% male, *p* = .011) and tended to have more fast‐repairing osteocytes as compared to cultures from young mice for both male and female groups, although the increased abundance of fast‐repairing osteocytes did not reach statistical significance (+57% female, +40% male, *p* = .12; Figure 2e).

**Figure 3:**
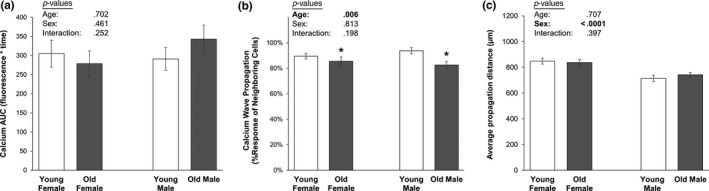
Calcium wave propagation initiated by a membrane disruption is impaired in aged osteocytes. (a) Influx of extracellular calcium induced by a membrane disruption was not different between old and young osteocytes in the wounded cell (*p* = .702). (b) Aged osteocytes were significantly impaired in their ability to initiate calcium signaling in neighboring, nonwounded osteocytes following creation of a membrane disruption (*p* = .006). No differences were observed between male and female cultures. *p < .05 versus sex‐matched young control. (c) Calcium wave propagation distance, measured from the PMD site to the center of the furthest cell demonstrating a calcium response, was not different between old and young groups, but propagation distance was greater in female as compared to male osteocyte cultures (*p* < .0001). *N* = 3 independent cell lines per age and sex

Accelerating PMD repair rates, while beneficial for enhancing postwounding cell survival (Hagan et al., 2019), can blunt downstream mechanotransduction: Fast repair rates limit the amount of ATP released from the wounded cell to initiate mechanotransduction in nonwounded neighboring cells (Mikolajewicz et al., 2019, 2018; Yu et al., 2018). To test whether the accelerated PMD repair rate observed in aged osteocytes affected mechanotransduction, we monitored calcium signaling initiated by a laser‐induced PMD, as previously described (Yu et al., 2018). While there were no differences in the magnitude of the calcium responses of the wounded cell between old and young osteocytes (Figure 3a), aged osteocytes were significantly impaired in their ability to initiate calcium signaling (i.e., propagate a calcium wave) in immediately adjacent nonwounded cells as compared to young osteocytes following creation of a PMD (Figure 3b). There were no differences in the distance of calcium wave propagation (*p* = .707, Figure 3c), although this may be due to the lack of flow stimulus or the small field of view (850 µm^2^). No interaction effects were observed between age and sex (*p* = .198, Figure 3b).

### 2.4 Osteocyte oxidative stress increases with aging, and impairing postwounding survival via oxidative stress leads to selective survival of fast‐repairing osteocytes

**Figure 4:**
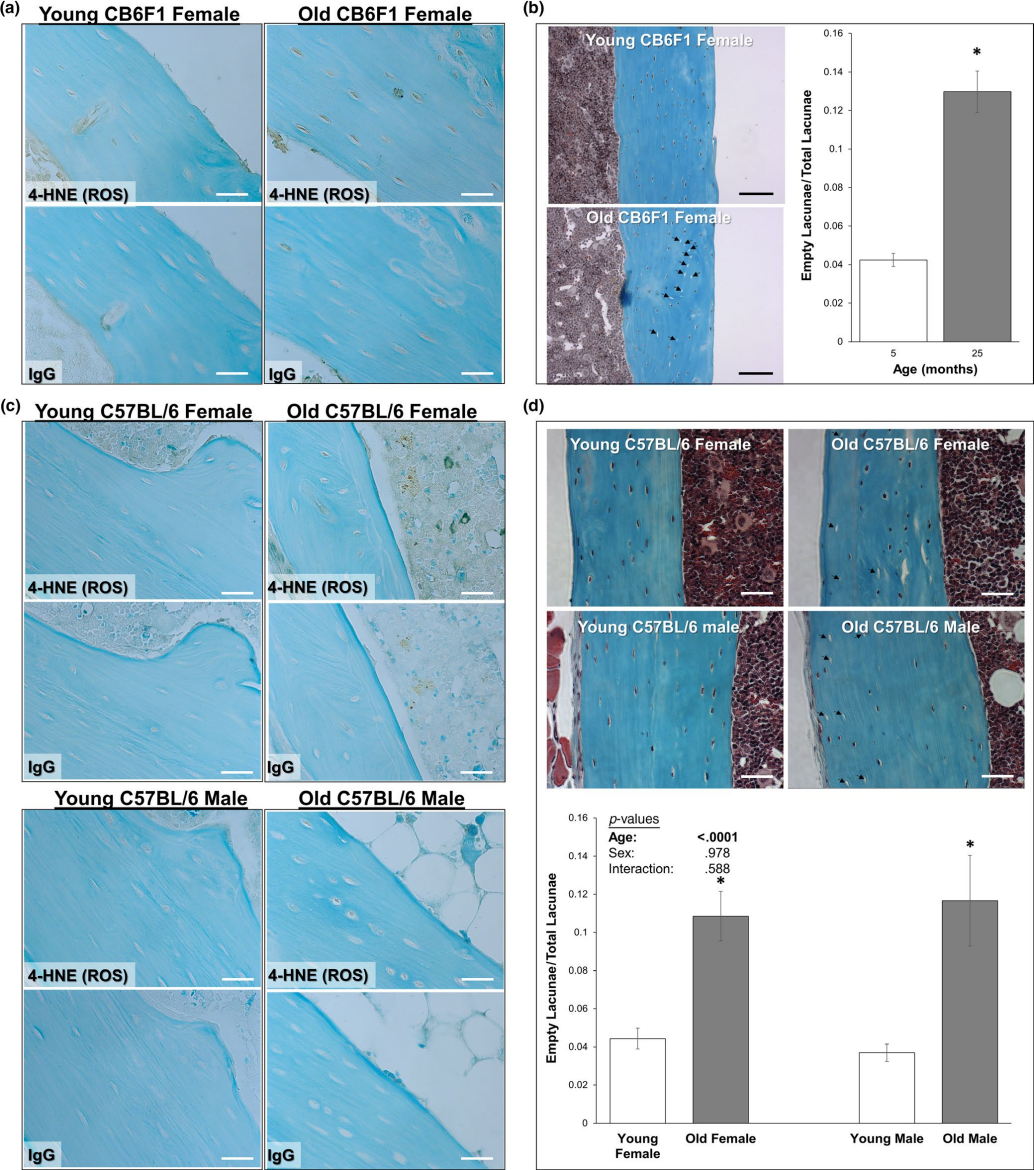
Aged osteocytes are exposed to greater oxidative stress in vivo. Longitudinal tibial sections of old and young mice used for the treadmill running experiments (a–b) and for isolation of primary osteocytes (c–d) were stained to detect 4‐HNE (brown staining, panels a and c), a marker of lipid peroxidation and oxidative stress. Representative samples from each group are shown. Little to no staining was observed with a nonspecific IgG control. Counterstain: Fast Green, 400× original magnification. Additional sections were stained with Goldner's Trichrome (panels b and d) for detection and quantification of empty osteocyte lacunae. Representative images are shown; empty osteocyte lacunae are highlighted by arrows. Scale bars: 30 µm (white), 100 µm (black). *p < .05 versus sex‐matched young control

**Figure 5:**
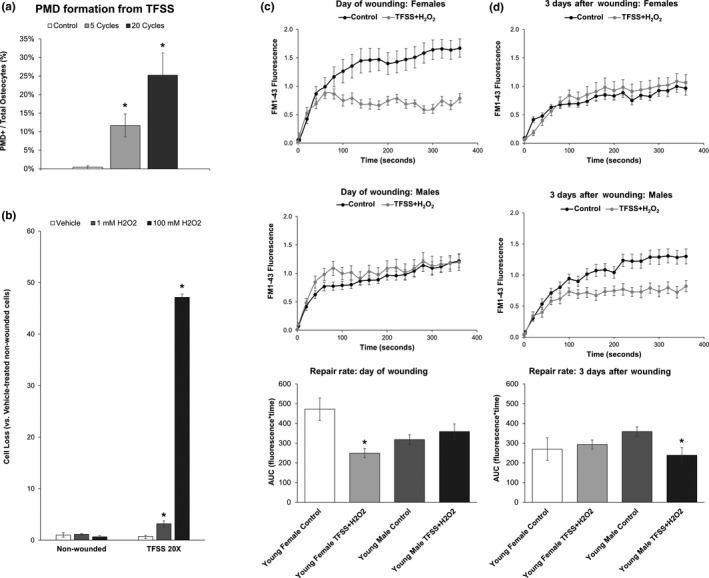
Selective survival of fast‐repairing osteocytes following turbulent fluid shear stress (TFSS) and oxidative stress. (a) MLO‐Y4 osteocytes were subjected to 5 or 20 cycles of TFSS, as described (Mikolajewicz et al., 2018), in the presence of 10 kDa fluorescein‐conjugated dextran. Control treated cells were subjected to one round of gentle media displacement in the presence of 10 kDa fluorescein‐conjugated dextran. *p < .05 versus control. (b) MLO‐Y4 osteocytes were subjected to 20 cycles of TFSS in the presence or absence of oxidative stress from hydrogen peroxide (1 mM H_2_O_2_). Cell death, as measured by comparison of quantified cell number before and after TFSS application (Panel b), was increased by the combination of TFSS + oxidative stress. *p < .05 versus nonwounded control, average of *n* = 3 independent experiments is shown. (c) Young female osteocytes treated with TFSS + H_2_O_2_ (1 mM) demonstrated faster rates of membrane repair as compared to control treated cells on the day of wounding, with significantly smaller area under the curve (AUC). Males, in contrast, showed no differences between groups on the day of wounding. (d) Young male osteocytes treated with TFSS + H_2_O_2_ demonstrated faster rates of membrane repair as compared to control treated cells, with significantly smaller area under the curve (AUC), when repair rate was measured three days after wounding. This trend was not seen in females. *p < .05 versus sex‐matched control group. *N* = 3 independent cell lines per sex

Oxidative stress impairs postwounding survival in osteocytes (Hagan et al., 2019) and has been reported to increase with age in bone (Almeida et al., 2007). Consistent with these observations, 4‐HNE staining (a marker of oxidative stress) was increased in the cortical bone osteocytes in long bones of old mice used for in vitro and in vivo experiments, along with an increased abundance of empty osteocyte lacunae in old mice, as previously reported (Tiede‐Lewis et al., 2017) (Figure 4). Given these results, and the surprising observation of faster repair rate in aged osteocytes (Figure 2), we hypothesized that fast‐repairing osteocytes may be naturally selected via progressive loss of slower‐repairing osteocyte populations to repair failure induced by oxidative stress. To test this, a turbulent fluid flow shear stress model was developed to facilitate wounding large numbers of osteocytes under sterile conditions. Application of 20 cycles of TFSS wounded approximately 25% of cells in dishes of MLO‐Y4 osteocytes (Figure 5a), and similar to previous studies using laminar fluid shear or bead wounding (Hagan et al., 2019; Yu et al., 2018), application of TFSS in the presence of H_2_O_2_ promoted postwounding cell death as compared to nonwounded cells (+3.1‐fold 1 mM H_2_O_2_, +47.1‐fold 100 mM H_2_O_2_; Figure 5b). TFSS was then applied to primary osteocytes from young mice in the presence or absence of H_2_O_2_, after which repair rate was quantified in the surviving cells. In males, repair rate of a laser‐induced plasma membrane disruption was not different between control and H_2_O_2_ + TFSS‐treated osteocytes on the same day of wounding (Figure 5c). Following a 3‐day incubation period, however, male osteocytes treated with H_2_O_2_ + TFSS demonstrated a significantly faster repair rate than control treated cells, as evidenced by area under the curve (Figure 5d). In females, H_2_O_2_ + TFSS‐treated osteocytes demonstrated a significantly faster repair rate than control treated cells on the day of wounding, but this trend did not persist when analyzed 3 days after wounding (Figure 5c–d).

From these observations, we propose that osteocyte PMD repair rate spans a wide spectrum of repair ability in the bones of younger subjects. With aging, we hypothesize that slower‐repairing osteocytes eventually succumb to repair failure and necrosis, perhaps due to repair‐inhibiting factors like oxidative stress, leading to a natural selection of fast‐repairing osteocytes with age (please see the Graphical Abstract as an illustration of these concepts).

## 3 DISCUSSION

As membrane repair may be impaired with age in other cells like myocytes (McBride, 2000), and osteocyte numbers decrease with age (Mullender et al., 1996; Tiede‐Lewis et al., 2017), we anticipated observing impaired PMD repair processes in old osteocytes. Surprisingly, however, aged osteocytes demonstrated less wounding with fluid flow shear stress (in vitro) or treadmill exercise (in vivo), and an enhanced PMD repair rate when wounding did occur as compared to osteocytes from younger animals. The increased rate of repair and the decreased abundance of slow‐repairing cells from aged mice suggest the possibility of a survival‐based selection process with aging: Osteocytes which repair more quickly may be more likely to survive repeated membrane wounding from physiological loading during the aging process, whereas slow‐repairing may be preferentially eliminated via repair failure (and consequently cell death) over time. This phenomenon would explain the observed increase in the ratio of faster‐repairing cells in the old osteocyte lines as compared to their younger counterparts, and the loss of osteocytes observed with aging both in this study (Figure 4) and in many previous publications (Mullender et al., 1996; Tiede‐Lewis et al., 2017).

While PMD formation in response to fluid shear was comparable between male and female osteocytes (Figure 1), we noted some significant differences in PMD repair processes between female and male osteocytes in the current study. In particular, female osteocytes tended to repair a PMD more quickly than male osteocytes (Figure 2a–d). Likewise, female osteocytes wounded in the presence of H_2_O_2_ suggested a selective loss of slower‐repairing osteocytes when tested on the same day of wounding (evidenced by a population‐wide enhancement of repair rate), whereas this trend was not apparent in male osteocytes until 3 days after wounding (Figure 5c–d). At present, the molecular mechanisms behind this sexually dimorphic response are not yet known. However, in ongoing studies, we are working to address the role of estrogen in PMD formation and repair via ovariectomy models. It will be important to determine whether estrogen plays a role in osteocyte PMD mechanisms, as it is well known that osteoporosis is more prevalent in females than in males due to the influence of hormonal changes associated with menopause on skeletal biology.

Plasma membrane disruptions develop with loading on the processes of osteocytes as fluid flow shear stress acts upon the cell (Yu et al., 2018), and studies presented here demonstrate that PMD formation was reduced in aged primary osteocytes and in aged bone. While we observed a decrease in osteocyte viability in aged bone, as has been previously reported, it is important to note that the magnitude of osteocyte PMD formation detected following loading in vivo should not have been impacted by this occurrence, as the number of wounded osteocytes detected was normalized to the population of viable osteocytes (intact nucleus as seen by DAPI staining). Moreover, the age‐related decrease in the percentage of osteocytes that developed load‐induced PMDs was also seen during in vitro experiments with viable primary osteocytes extracted from the bone tissue. The decreased number of PMD formed with loading in aged primary osteocytes and in aged bone may be influenced, at least in part, by changes in cell morphology. Recent reports indicate that osteocyte dendrite number and length are decreased in aged bone from both humans and mice (Milovanovic et al., 2013; Tiede‐Lewis et al., 2017). Studies presented here are consistent with these observations and suggest that these morphological changes may contribute to the decreased PMD formation with loading in aged bone, as an osteocyte with fewer (females) or shorter (males) processes may be less likely to develop a PMD with loading. A second possible explanation for the decreased osteocyte PMD formation in aged bone relates to the microenvironment of pericellular matrix surrounding the cell. Previous reports indicate that drag forces on the osteocyte pericellular matrix are important for osteocyte mechanosensation, and that the volume fraction of pericellular matrix decreases with age (Lai et al., 2015; Price, Zhou, Li, & Wang, 2011; Thompson et al., 2011; Wang et al., 2014). Our data are consistent with these findings and support the hypothesis that drag forces on pericellular matrix are important for the formation of osteocyte PMD with loading, since both PMD formation and pericellular matrix production were reduced in the aged osteocytes. Moreover, this phenotype could be recapitulated in immortalized osteocytes, where PMD formation was reduced in MLO‐Y4 cells by degrading the osteocyte pericellular matrix with hyaluronidase, which also blunts downstream mechanotransduction (Reilly et al., 2003). The present study also suggests that the reduced pericellular matrix associated with aged osteocytes occurs in a cell‐intrinsic fashion, as aged primary osteocytes secreted less pericellular matrix in vitro as compared to younger cells. A weakness of our study is that due to technical limitations, pericellular matrix staining was only conducted in male cell lines. However, we are encouraged by the fact that when cells from both sexes were available, they responded similarly (i.e., PMD abundance with fluid flow shear stress).

The most surprising finding from the current study is the observation of enhanced repair rate in aged as compared to young osteocytes. In contrast to osteocytes, membrane repair processes can be impaired with aging in other tissues like muscle. For example, resting membrane potential takes longer to repolarize to control values in aged as compared to young–adult muscles, and aged muscles exposed to a second eccentric contraction (most damaging) demonstrate a significantly reduced adaptive response (T. McBride, 2000). Similarly, previous studies have suggested that muscles from older animals are more susceptible to mechanical damage from contraction‐induced injury (Brooks & Faulkner, 1996); when subjected to the same eccentric loading protocol, muscles from old mice show a greater degree of injury, and when subjected to the same level of damage, muscle fibers from old animals show impaired recovery as compared to young animals (Brooks & Faulkner, 1990; Zerba, Komorowski, & Faulkner, 1990). In contrast, aged osteocytes studied here demonstrated enhanced repair rates and less evidence of the formation of plasma membrane disruptions with loading as compared to osteocytes from young mice. Faster PMD repair rate, while beneficial for cell survival, may have deleterious consequences for downstream osteocyte mechanotransduction. In our first report on osteocyte PMD and mechanotransduction, we noted that accelerating PMD repair rate via antioxidant treatment actually blunted the downstream activation of osteocyte mechanotransduction (Yu et al., 2018). Accordingly, the increased abundance of fast‐repairing osteocytes found in aged bone may contribute to the poor skeletal adaptation responses of bone in older individuals (Ireland, Maden‐Wilkinson, Ganse, Degens, & Rittweger, 2014; Kontulainen et al., 2003; Patel, Brodt, & Silva, 2014). If the majority of surviving osteocytes in aged bone repair at an accelerated rate compared to a more moderate repair rate of osteocytes from young bone, the cells may be repairing too quickly for mechanotransduction to be properly initiated. Data presented here support this interpretation, as aged osteocytes (with faster PMD repair rates) were impaired in their ability to propagate a mechanotransduction‐related calcium wave to neighboring, nonwounded osteocytes (Figure 3b). We acknowledge that while statistically significant, the absolute effect of age on calcium wave propagation was mild. It is possible that other metrics of mechanotransduction propagation not measured here, such as ATP release by the wounded cell, could show greater absolute changes with aging (Mikolajewicz et al., 2019, 2018); this prospect will be investigated in future studies. Still, following the logic supported by selective survival of fast‐repairing osteocytes and poor calcium wave propagation by those same cells, the impaired adaptation of aged bone to mechanical loading may be driven in part by surviving osteocytes possessing rapid PMD repair rate which blunts their ability to properly communicate mechanotransduction signals.

It is intriguing that impaired adaptation of aged bone can be rescued by “priming the system” through application of a short‐term, high magnitude bout of loading (Javaheri et al., 2018). As osteocyte PMD form preferentially with high‐impact loading (Yu et al., 2018), this reported rescue of aged bone mechanosensitivity may be consistent with a PMD‐based mechanism of osteocyte mechanosensation and rapid PMD repair in aged osteocytes, suggesting aged osteocytes may require more substantial membrane damage to trigger an appropriate downstream mechanotransduction response. A recent study found that PMD formed by loading in bone cells are on the order of 5 nm in size (Mikolajewicz et al., 2018), but the effects of aging on osteocyte PMD size are not yet known. Future experiments will explore these mechanisms in primary osteocytes exposed to fluid shear and laser‐based membrane wounding. The molecular pathways promoting enhanced repair in aged osteocytes are also the topic of ongoing studies, aimed to identify pathways that can be targeted to alter PMD‐initiated mechanotransduction. Targeting slow‐repairing osteocytes in young subjects to enhance osteocyte viability during aging or targeting fast‐repairing osteocytes in aged subjects to improve mechanotransduction may represent novel targets for therapeutic intervention in degenerative bone diseases.

## 4 EXPERIMENTAL PROCEDURES

### 4.1 Animals

All experiments followed NIH guidelines and were approved by the Institutional Animal Care and Use Committee at Augusta University. Male and female C57BL/6 mice were obtained from the NIA aged rodent colony (*n* = 5 per sex and age; 3 or 24 months old) and a commercial supplier (Charles River; *n* = 5 per sex, 3 months old). Old and young female mice from a hybrid genetic background (CB6F1; BALB/cBy x C57BL/6; *n* = 5 per age, old = 24 months, young = 4 months) were obtained from the NIA for treadmill running experiments, as preliminary experiments confirmed poor treadmill running ability in the C57BL/6 mice as previously reported (Lerman et al., 2002) (data not shown). Female mice were specifically chosen for in vivo treadmill running experiments to avoid confounding influence from fighting in group‐housed male mice (Meakin et al., 2013), although we have previously observed that osteocyte PMD occur with treadmill loading in both male and female mice (Hagan et al., 2019; Yu et al., 2018). All mice were housed in standard rodent cages with a 12‐hr light/ 12‐hr dark schedule. Mice were permitted water and standard rodent chow (RD: Teklad #2918) ad libitum.

### 4.2 Primary osteocyte isolation and culture

Primary osteocytes were isolated from long bone diaphyses (femur, tibia, and humerus) from five mice of each age and sex according to published methods (Stern et al., 2012). Briefly, long bones (two femurs, two humerii, and one tibia per mouse) were harvested aseptically, after which epiphyses were removed and marrow was flushed from the diaphysis. The remaining tibia from each mouse was fixed in 10% neutral buffered formalin for histology. The flushed cortical bone shafts from all mice in each group were collected and pooled, then separated into three separate batches (*n* = 8 to 9 diaphyses each) for cell line isolation. Bone shafts were subjected to serial digestions with collagenase (Type 1A, Sigma‐Aldrich #C9891) and EDTA, and cells from digests 7 through 9 along with cells from bone chip outgrowth were pooled and considered to be an osteocytic population, as described (Stern et al., 2012). Cells were plated onto type 1 collagen‐coated dishes in osteocyte culture medium [α‐MEM (Invitrogen) + 5% fetal bovine serum (FBS, Atlanta Biologicals) + 5% bovine calf serum (HyClone) + 1% antibiotic/antimycotic)] and grown to 70% confluency, at which time cells were re‐seeded for all subsequent experiments. All primary osteocyte experiments were conducted within 2 weeks of isolation, as recommended (Stern et al., 2012).

### 4.3 Primary osteocyte morphology

Osteocytes were seeded into 60‐mm dishes in osteocyte culture medium and imaged with a 10× objective 4 days after plating. Cell morphology including cell area, number of dendrites per cell, and dendrite length was quantified for 2–3 images collected at randomized locations in the dish for each cell line (*n* = 3 lines per age and sex) using image analysis software (Bioquant Osteo). At least five cells were measured in each image.

### 4.4 Laser wound repair rates in primary osteocytes

Osteocytes were seeded into 60‐mm dishes in osteocyte culture medium and wounded with a multiphoton laser in PBS containing calcium (1.8 mM) and FM1‐43 dye (3 µM), as we previously described (Yu et al., 2018). One PMD per osteocyte, located on a dendritic process, was created. FM1‐43 dye influx (measured as area under the curve, AUC) was used to quantify PMD repair as previously described (Howard, McNeil, & McNeil, 2011; Yu et al., 2018). The derivative of the FM1‐43 fluorescence versus time curve was calculated up to 60 s after wounding to permit analysis of the curve slope, indicative of repair rate. Conceptually, steep slopes (indicative of large amounts of dye entering the cell quickly) correspond with slower PMD repair rates, whereas more gradual slopes (indicative of curve plateau from inhibited dye entry) correspond with faster PMD repair rates. The AUC of this PMD repair rate curve for each sample was calculated as described above. In addition, the slope of the curve at 60 s after PMD formation was used to group cells into one of three categories: fast, medium, or slow repair rate. The upper and lower quartiles for repair rate at 60 s after wounding were calculated for all datasets, and “fast‐repairing” cells were defined as those with a curve slope less than the lower quartile of the data. Likewise, “slow‐repairing” cells were defined as those with a curve slope greater than the upper quartile of the data. Cells with repair rates falling within the interquartile range were defined as having a “medium” repair rate.

### 4.5 Calcium signaling in primary osteocytes

Osteocytes were loaded with Cal‐520‐AM dye, after which calcium signaling was initiated by a laser‐induced PMD as described (Yu et al., 2018). Fluorescence was measured every second and normalized to baseline (pre‐PMD) fluorescence (F/Fo) both in the wounded cell and in the nonwounded neighboring osteocytes. Calcium signal intensity was quantified in the wounded cells as the area under the fluorescence versus time curve. Calcium signal propagation was defined and quantified as the percentage of nonwounded neighboring cells exhibiting a temporal increase in fluorescence that was at least five times higher than background fluorescence. The distance (in microns) of calcium wave spread to nonwounded adjacent cells was also calculated from the site of the laser PMD to the center of the furthest cell showing a calcium response as defined above.

### 4.6 Fluid flow shear stress, parallel plate flow chamber

Osteocytes were seeded into type 1 collagen‐coated flow chamber slides (Ibidi, µSlide VI^0.4^, #80606; 1,000 cells per channel) and cultured for 4 days with fresh media added daily. Cells were subjected to fluid flow shear stress (30 dynes/cm^2^) for 5 min using a syringe pump (Harvard Apparatus PHD Ultra I/W) and culture medium supplemented with 1 mg/ml of 10 kDa fluorescein‐conjugated dextran used as a membrane disruption tracer, as previously described (Yu et al., 2018). At the conclusion of experiments, cells were washed 3 times with PBS and imaged on a confocal microscope (Zeiss). Uptake of fluorescein dextran was interpreted as evidence of a membrane disruption event; wounded cells permit entry of the dextran molecule, and successful PMD repair seals the tracer inside the cell (Yu et al., 2018). The percentage of wounded osteocytes was quantified in each experiment. A total of 10 to 20 pictures were collected and analyzed per cell line.

### 4.7 Turbulent fluid shear with oxidative stress

To determine whether postwounding cell death from oxidative stress affected the average membrane repair rate of an osteocyte population, primary osteocytes from young mice (*n* = 3 cell lines per sex) were treated with 1 mM H_2_O_2_ immediately prior to application of 20 cycles of turbulent fluid shear stress (TFSS), generated by media displacement as recently described (Mikolajewicz et al., 2018). Control dishes were subjected to one round of gentle media displacement (using the slowest setting on the pipet aid, with liquid displacement occurring at the edge of the dish). Pilot experiments with MLO‐Y4 osteocytes demonstrated that 20 cycles of TFSS generated plasma membrane disruptions in 25% of the cells in the dish (as demonstrated by uptake of 10 kDa fluorescent dextran; Figure 5a), and that cell death selectively occurred with the combination of TFSS + H_2_O_2_ (as measured by microscopic quantification of cell loss (Figure 5b)). Immediately after treatment, the primary osteocytes were incubated for 10 min at 37 deg C and then gently washed three times in PBS with 1.8 mM Ca^2+^. Cells were then either subjected immediately to repair rate analysis with FM 1–43 dye as described above or incubated in culture medium for three days prior to repair rate analysis.

### 4.8 Pericellular matrix staining

Osteocytes (male cell lines only due to cell number limitations) were seeded onto glass coverslips coated with type 1 collagen and allowed to grow for 4 days. Cells were fixed in 4% paraformaldehyde and stored in PBS, followed by staining with a lectin wheat germ agglutinin‐FITC conjugate (WGA‐FITC; GeneTex, Irvine CA #GTX01502) to detect pericellular matrix as previously described (Barker et al., 2004; Reilly et al., 2003). Briefly, coverslips were stained with 1 µg/ml WGA‐FITC in PBS for 15 min, washed with PBS, and mounted with medium containing DAPI (Vectashield, Vector Laboratories). Cells were imaged on a confocal microscope (Zeiss), and 15 cells were imaged at random per cell line (*n* = 3 independent cell lines for young, three for old) using a 63× oil immersion objective. The intensity of the FITC signal was measured and averaged between two circular regions of interest (10 µm diameter) per cell in the cell body using the manufacturer's software (Zeiss).

### 4.9 Treadmill exercise

Mice were allowed to acclimate to the animal facility for 3 weeks after arrival. One week prior to experiments, mice were trained to run on the treadmill at 10 m/min for 10 min/day on three consecutive days. One aged mouse died during these training sessions from unknown causes. To determine the effects of acute loading (one exercise bout) on osteocytes, the remaining CB6F1 mice (*n* = 5 young, four old) were subjected to one strenuous exercise bout (−15° downhill, linearly increasing speed from 10 m/min to 20 m/min over 30 min). All mice received intraperitoneal injections of Evans Blue (50 mg/kg body mass) to act as an exogenous PMD tracer 18 hr prior to the exercise bout. Mice were run in old/young pairs, and exercise was stopped when one mouse reached fatigue. In each trial, the old mouse consistently fatigued first after approximately 20 min of running. All mice were sacrificed within 2 hr of loading. Mice were perfused with 10% formalin prior to tissue collection.

### 4.10 Detection of plasma membrane disruptions in cortical bone

Plasma membrane disruptions were detected in histological sections by detection of an exogenous (Evans Blue) or endogenous (serum albumin) membrane disruption tracer, as previously described (Yu et al., 2018). The right femur from each mouse used in the treadmill studies was fixed in 10% neutral buffered formalin, cryosectioned with cryofilm IIC, stained with FM 1–43 for labeling of intracellular membranes, and imaged on a multiphoton microscope (Zeiss) with a 20X dipping objective to detect osteocytes labeled with intracellular Evans Blue as previously described (Hagan et al., 2019; Yu et al., 2018). To validate these measurements with an endogenous PMD tracer, the left tibia from each mouse was fixed in 10% neutral buffered formalin, decalcified with EDTA, paraffin‐embedded, and stained with a FITC‐conjugated mouse albumin antibody (AIFAG3140, Accurate Chemical Corp.) for detection of intracellular albumin using an inverted confocal microscope (Zeiss) (Hagan et al., 2019). For either protocol, a Z‐stack series of 20–30 images were collected through section thickness and processed into a maximum intensity projection. Five to ten images per bone were captured and analyzed with Bioquant OSTEO software to quantify the percentage and spatial density of PMD‐labeled osteocytes (Yu et al., 2018).

### 4.11 Lacunar occupancy and oxidative stress

Paraffin‐embedded tibial sections from the CB6F1 mice used in treadmill studies and the C57BL/6 mice used for primary osteocyte isolation studies were stained with Goldner's Trichrome to determine osteocyte lacunar vacancy as a measure of cell viability as previously described (Hagan et al., 2019). Bone sections were examined at 200X magnification, and a total of 10 images per bone were collected at random locations throughout the diaphysis. The percentage of empty osteocyte lacunae was quantified with image analysis software (Bioquant OSTEO). Additional tibial sections were subjected to 4‐HNE staining to detect osteocyte lipid peroxidation as an indicator of oxidative stress, as we recently described (Hagan et al., 2019); staining with a nonspecific IgG control was utilized to control for any off‐target background staining. Bones were imaged with at 400X with an oil immersion objective.

### 4.12 MLO‐Y4 fluid flow shear stress experiments

MLO‐Y4 cells, courtesy of Dr. Lynda Bonewald, were maintained in growth medium (α‐MEM (Invitrogen) + 5% fetal bovine serum (FBS, Atlanta Biologicals) + 5% bovine calf serum (HyClone) + 1% penicillin/streptomycin). Cells were seeded into type 1 collagen‐coated flow chambers and cultured for 4 days as described in the “Fluid Flow Shear Stress” section above. Immediately before experiments, cells were treated with either vehicle or hyaluronidase (*Streptomyces hyalurolyticus*, Sigma‐Aldrich #H1136) at a final concentration of 160 U/ml in serum‐free alpha MEM for 1 hr at 37 degrees C to remove pericellular matrix as previously described (Reilly et al., 2003). After treatment, cells were washed with PBS and subjected to 5 min of 30 dynes/cm^2^ fluid flow shear stress in culture medium containing 10 kDa fluorescein‐conjugated dextran for detection of membrane disruptions as described above.

### 4.13 Statistics

For each property, groups were compared by *t* tests (acute treadmill exercise: 2 Age; MLO‐Y4: 2 Treatment) or 2‐factor ANOVA with interaction (2 Age × 2 Sex) followed by Tukey's post hoc analyses when appropriate using JMP 14.0 (SAS Inc., Cary, NC). For repair rate analysis in young osteocytes following TFSS, groups were compared with *t* tests (two Treatment). Statistical significance was set at *p* < .05 for all comparisons. For quantification of histological/imaging studies, data were collected in a blinded fashion, and the same operator collected all data within a given experiment to limit interobserver variability. Data are presented as mean ± standard error (*SE*), unless otherwise indicated. Sample sizes are indicated in each figure and/or caption.

## CONFLICT OF INTEREST

None declared.

## AUTHOR CONTRIBUTIONS

MLH, LW, CMI, MWH, PLM, and MEML designed the study. MLH, KY, JZ, BNV, and RLR collected the data. MLH, MHJ, LW, CMI, MWH, PLM, and MEML interpreted the data. MLH, CMI, MWH, and MEML drafted the manuscript. All authors approved the final version of manuscript.

## Data Availability

The datasets supporting the conclusions of this article are available from the corresponding author upon reasonable request.
