# Supplementary figures and images for: Decreased pericellular matrix production and selection for enhanced cell membrane repair may impair osteocyte responses to mechanical loading in the aging skeleton
Source: Aging Cell. 2019 Nov 19;19(1):e13056. doi: 10.1111/acel.13056 (PMC6974724; doi:10.1111/acel.13056)

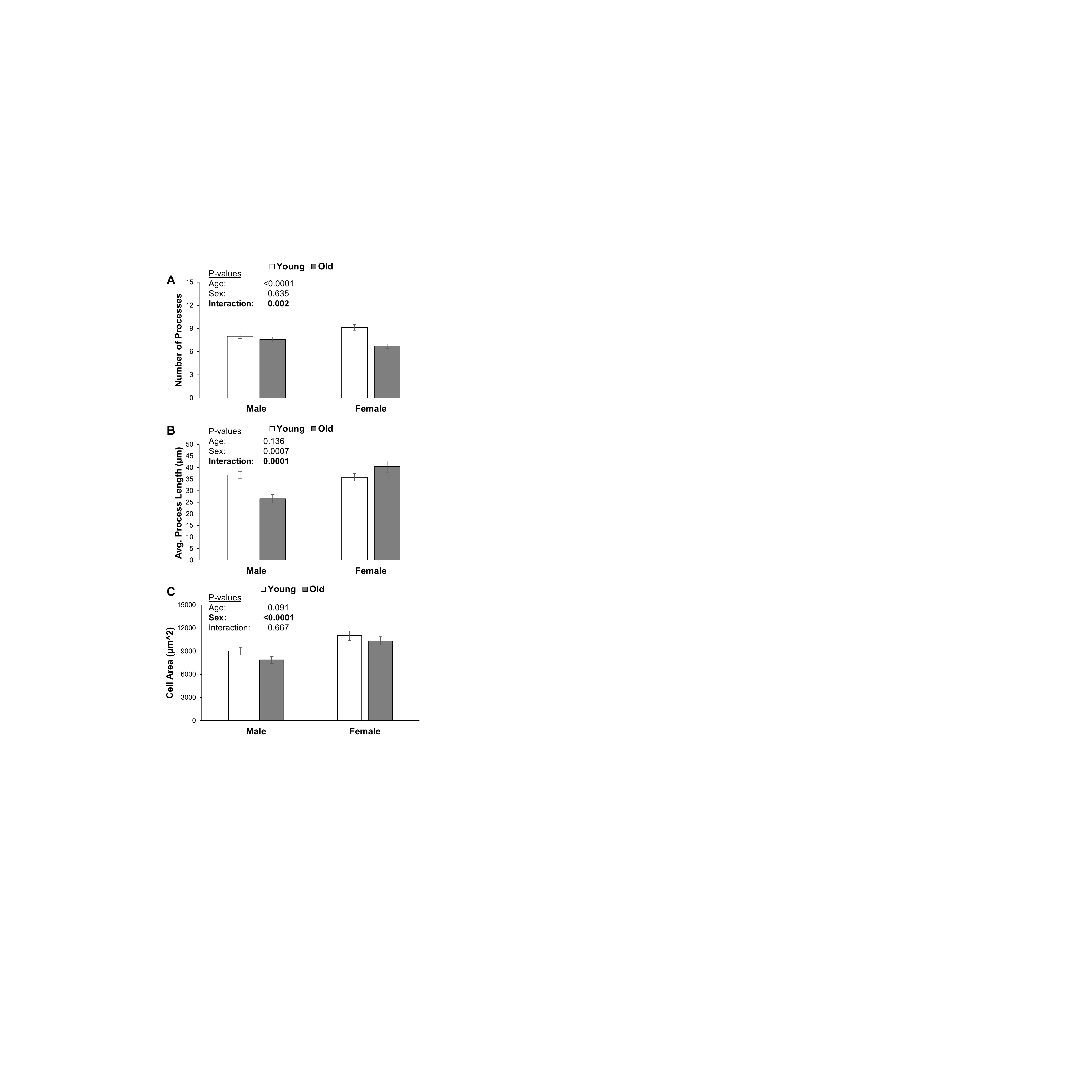

Supplement: Supplementary file 1 [file ACEL-19-e13056-s001.TIF]
